# Suppressing postcollection lysophosphatidic acid metabolism improves the precision of plasma LPA quantification

**DOI:** 10.1016/j.jlr.2021.100029

**Published:** 2021-01-30

**Authors:** Kuniyuki Kano, Hirotaka Matsumoto, Nozomu Kono, Makoto Kurano, Yutaka Yatomi, Junken Aoki

**Affiliations:** 1 Department of Health Chemistry, Graduate School of Pharmaceutical Sciences, University of Tokyo, Bunkyo-ku, Tokyo, Japan; 2 Laboratory of Molecular and Cellular Biochemistry, Graduate School of Pharmaceutical Sciences, Tohoku University, Aoba-Ku, Sendai, Japan; 3 AMED-LEAP, Japan Science and Technology Corporation, Kawaguchi, Saitama, Japan; 4 Department of Clinical Laboratory, University of Tokyo Hospital, Bunkyo-ku, Tokyo, Japan

**Keywords:** lysophosphatidic acid, plasma, clinical specimen, metabolism, autotaxin, LC-MS, biomarker, ATX, autotaxin, CTAD, citrate-theophylline-adenosine-dipyridamole, LPA, lysophosphatidic acid, LPC, lysophosphatidylcholine, LPL, lysophospholipid, lysoPLD, lysophospholipase D, PC, phosphatidylcholine, PPP, platelet-poor plasma, PRP, platelet-rich plasma

## Abstract

Lysophosphatidic acid (LPA) is a potent signaling lipid, and state-dependent alterations in plasma LPA make it a promising diagnostic marker for various diseases. However, plasma LPA concentrations vary widely among reports, even under normal conditions. These variations can be attributed, at least in part, to the artificial metabolism of LPA after blood collection. Here, we aimed to develop an optimized plasma preparation method that reflects the concentration of LPA in the circulating blood. The main features of the devised method were suppression of both LPA production and degradation after blood collection by keeping whole blood samples at low temperature followed by the addition of an autotaxin inhibitor to plasma samples. Using this devised method, the LPA level did not change for 30 min after blood collection. Also, human and mouse LPA levels were found to be much lower than those previously reported, ranging from 40 to 50 nM with minimal variation across the individual. Finally, the increased accuracy made it possible to detect circadian rhythms in the levels of certain LPA species in mouse plasma. These results demonstrate the usefulness of the devised plasma preparation method to determine accurate plasma LPA concentrations.

Lysophosphatidic acid (LPA: 1- or 2-acyl-sn-glycerol-3-phosphate) is a bioactive lipid that is pleiotropic with many biological functions. LPA exerts its biological effects by binding to and activating six G-coupled receptors (LPA_1_–LPA_6_) (1, 2). LPA is present in various body fluids, including blood (serum and plasma), cerebrospinal fluid, seminal plasma, and saliva (3, 4, 5, 6). Several clinical studies have reported elevated plasma LPA levels in patients with various diseases, including malignant tumors (7, 8), hepatitis (9), and acute coronary syndrome (ACS) (10, 11), raising the possibility that plasma LPA could be used as a biomarker for these diseases. Although plasma LPA levels are clinically important, their accuracy is controversial because reported values, even under normal conditions, can vary by orders of magnitude. For example, plasma LPA levels varied widely in both patients with ovarian cancer (1.0–43.1 μM) and in healthy controls (0.1–6.3 μM) (12). In addition, LPA concentrations in normal human subjects have been reported at 200 nM (13), 120 nM (14) and several tens of nanomoles (15). The analyses have shown a large scattering even in the same test, suggesting the difficulty of handling blood samples for precise LPA quantification.

In circulating blood, LPA is continually produced and degraded. As for LPA production, divalent cation-sensitive autotaxin (ATX) is present in plasma and converts lysophospholipids (LPLs), mainly lysophosphatidylcholine (LPC), to LPA by its lysophospholipase D activity (16). In fact, in incubated plasma, the LPA concentration increased in a time-dependent manner to the level of micromoles within an hour (17). Curiously, however, LPA concentrations in freshly prepared plasma (and probably in circulating blood) are much lower. This discrepancy is explained by the fact that LPA in the blood is also continually degraded possibly by membrane-bound lipid phosphate phosphatases present on various blood cells and endothelial cells (18, 19). Presumably, in circulating blood, the balance of LPA production and degradation makes the LPA level constant. Thus, for precise LPA measurement, it is important to keep the balance during blood sampling, especially during plasma preparation. Previously, we examined the effect of anticoagulants and temperature on the production of LPA and LPC that occurs in the course of plasma preparation (14). We found that the use of two anticoagulants [EDTA (7.5 mM) and citrate-theophylline-adenosine-dipyridamole (CTAD, 10% (v/v))] in combination with low temperature (4°C) significantly lowered LPA and LPC formation. Under these conditions, LPA concentrations in normal human plasma samples were about 100–300 nM. The study used EDTA to inhibit the ATX activity. However, inhibition of ATX activity by EDTA is partial, depending on the concentration of the reagent. Recently, several ATX inhibitors have been developed (20, 21, 22), which can be used to suppress artifactual LPA formation in biological samples including plasma (15). However, the preceding reports including Yagi *et al.* (15) have not taken account of the fact the LPA is continually degraded during the blood sample handling and plasma preparation.

In this study, we applied an ATX inhibitor for precise LPA measurement. Of interest, however, we noticed that inhibition of LPA production by the ATX inhibitor resulted in an artifactual decrease in the LPA level, showing that the balance between the production and the degradation of LPA must be kept during preparation of the plasma. Accordingly, we tried to develop a plasma preparation method for precise LPA measurement by taking the balance into account. The resulting validated method revealed that accurate plasma concentration of LPA is about 40–60 nM, which is much lower than that expected from the previous studies. Using the method, we report a daily fluctuation of plasma LPA in mice for the first time.

## Materials and methods

### Reagents

All phospholipids except for LPA 22:6 were purchased from Avanti Polar lipids. LPA 22:6 was chemically synthesized, as previously described (23). Other chemicals were purchased from Wako Pure Chemical Industries unless otherwise indicated. ATX inhibitor (ONO-8430506) was kindly donated by ONO pharmaceutical company.

### Mice

C57BL/6J mice were purchased from SLC Japan. Mice were housed under specific pathogen-free conditions in an air-conditioned room on a light-dark cycle of 12 h each and fed standard laboratory chow ad libitum. The time of day is expressed in zeitgeber time (ZT). Lights are turned on at ZT0 and turned off at ZT12. All mice were treated in accordance with the procedure approved by the Animal Ethics Committee of the Graduate School of Pharmaceutical Sciences, Tohoku University, Japan.

### Sample preparation

In mice, whole blood was collected using heparinized hematocrit tube (Drummond, Philadelphia, PA) and then immediately mixed with EDTA (1 mg/ml) and ONO-8430506 (10 μM) as indicated. In human, blood was collected from an antecubital vein of male volunteers (21–24 years old, fasted over 12 h) who had not received any medication using EDTA vacutainer tube (NP-EN0507, NIPRO, Osaka, Japan) or Heparin vacutainer tube (VP-H050K, TERUMO, Tokyo, Japan). To obtain plasma, blood was centrifuged at 1,500 *g* for 5 min at 4°C. As soon as centrifugation was completed, the supernatant was collected and then immediately mixed with nine-volume of methanol containing internal standard (IS). In some experiments, whole blood and plasma were kept at room temperature or on ice. Methanol extraction is by far the best in extracting lysophospholipids including LPA (24). The extraction yield using methanol from plasma is 99.4% in LPA 18:1 (25). All samples were stored at –20°C until LC-MS/MS analysis.

### LC-MS/MS

Sample preparation and LC-MS/MS analysis were performed according to previously described methodology (25), with minor modifications. The methanol extract (10–20 μl) was subject to an autosampler of Ultimate3000 (Thermo Fisher Scientific, Tokyo, Japan) equipped with a C18 CAPCELL PAK ACR column (1.5 × 250 mm; Osakasoda, Osaka, Japan) for LPLs or C8 CAPCELL PAK UG120 column (1.5 × 250 mm; Osakasoda, Osaka, Japan) for phosphatidylcholine (PC). LC separation was performed with a gradient elution of solvent A (5 mM ammonium formate in water) and solvent B (5 mM ammonium formate in 95% (v/v) acetonitrile). The eluate was sequentially ionized using an ESI using a TSQ Quantiva or TSQ-Altis triple quadrupole mass spectrometer (Thermo Fisher Scientific, Tokyo, Japan). The multiple reaction monitoring transition from [M+H]^+^ to *m/z* 184 was selected for PC analysis. The multiple reaction monitoring analysis of LPLs was performed according to previously described methodology (24). The concentrations of each LPA species (LPA 16:0, 18:1, 18:2, 20:4, and 22:6) were determined using the corresponding LPA standards. Other lysoPL and PL concentrations were estimated from the area ratio to the internal standard (0.1 μM LPA 17:0, 1 μM LPC 17:0, and 3 μM PC 24:0). Under the present condition using TSQ-Altis triple quadrupole MS (Thermo Fisher scientific), the value of the lowest level of quantification is about 50 pM. We confirmed that all data in this study are within the quantitative range.

### Measurement of lysophospholipase activity

Lysophospholipase D (lysoPLD) activity was measured as described previously (26). Briefly, plasma samples were mixed with LPC 14:0 (100 mM Tris-HCl, 5 mM MgCl_2_, 500 mM NaCl, 0.05% Triton X-100, pH 9.0) and incubated for 5–9 h at 37°C. Liberated choline was quantified using choline oxidase, peroxidase, and TOOS reagent. The activity was indicated by the generation rate of choline per unit time and volume (pmol/ml/h).

### Data analysis

All data were analyzed using GraphPad Prism 8 software. To calculate statistical significance, one-way ANOVA followed by Dunnett's or Tukey multiple comparison test and two-way ANOVA with the Geisser-Greenhouse correction followed by Dunnett's multiple comparison test were used. A value of *P* < 0.05 was considered statistically significant. No sample size calculation was performed.

## Results and Discussion

### Plasma preparation methods for accurate quantitation of LPA

**Fig. 1:**
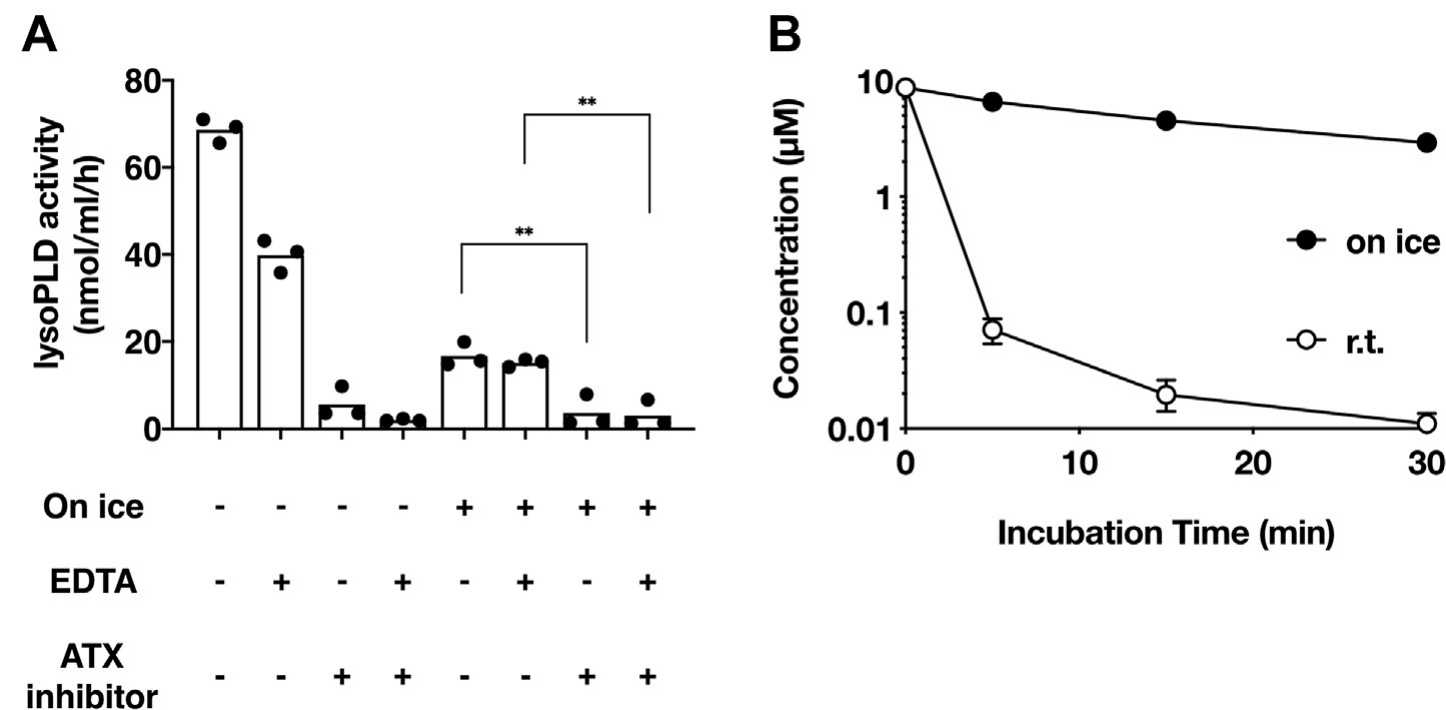
Effect of temperature and an ATX inhibitor on LPA metabolism. A: Effect of temperature and an ATX inhibitor on lysophospholipase D activity. LysoPLD assay was performed using heparinized mouse plasma as an enzyme source and 14:0-LPC as a substrate at room temperature (r.t.), on ice, and in the presence of EDTA (1 mg/ml) and the ATX inhibitor ONO-8430506 (10 μM). The lysoPLD activity was determined by quantifying released choline. The bars represent the mean of three replicas, and the individual values are shown as symbols. ∗∗*P* < 0.01, ordinary one-way ANOVA followed by Dunnett's multiple comparison test. B: Effect of temperature on LPA degradation in mouse whole blood. Mouse plasma was added with oleoyl-LPA (final 10 μM) and incubated at r.t. or on ice. The remaining oleoyl-LPA at the indicated time was determined by LC-MS/MS. Data are shown as mean ± SE (n = 4).

We first explored the conditions under which both LPA production and degradation were suppressed. For inhibition of LPA production, we utilized an ATX inhibitor ONO-8430506, EDTA, and low-temperature conditions (on ice). For inhibition of LPA degradation, we only utilized low-temperature (on ice) condition, since specific chemicals that inhibit the LPA degradative enzymes are not available. The lysoPLD activity of plasma ATX was almost completely and partially suppressed by ONO-8430506 and EDTA, respectively (Fig. 1A). In addition, the activity was also considerably suppressed on ice (Fig. 1A). It should be noted here that a slight lysoPLD activity was still detected even in the low-temperature condition both in plasma with and without EDTA in the absence of ONO-8430506. To evaluate the LPA degradative activity, we added LPA 18:1 (10 μM final), a minor LPA species in mouse plasma, to the whole mouse blood samples, and time-dependent LPA degradation was monitored by quantifying the remaining LPA 18:1 in the plasma. We found that LPA 18:1 was degraded quickly at room temperature (r.t.) but very slowly on ice (Fig. 1B). Thus, inhibition of LPA production and degradation by low temperature (on ice) appeared to be effective at keeping the LPA level constant in addition to the inhibition of LPA production by ATX inhibitors and EDTA.

**Fig. 2:**
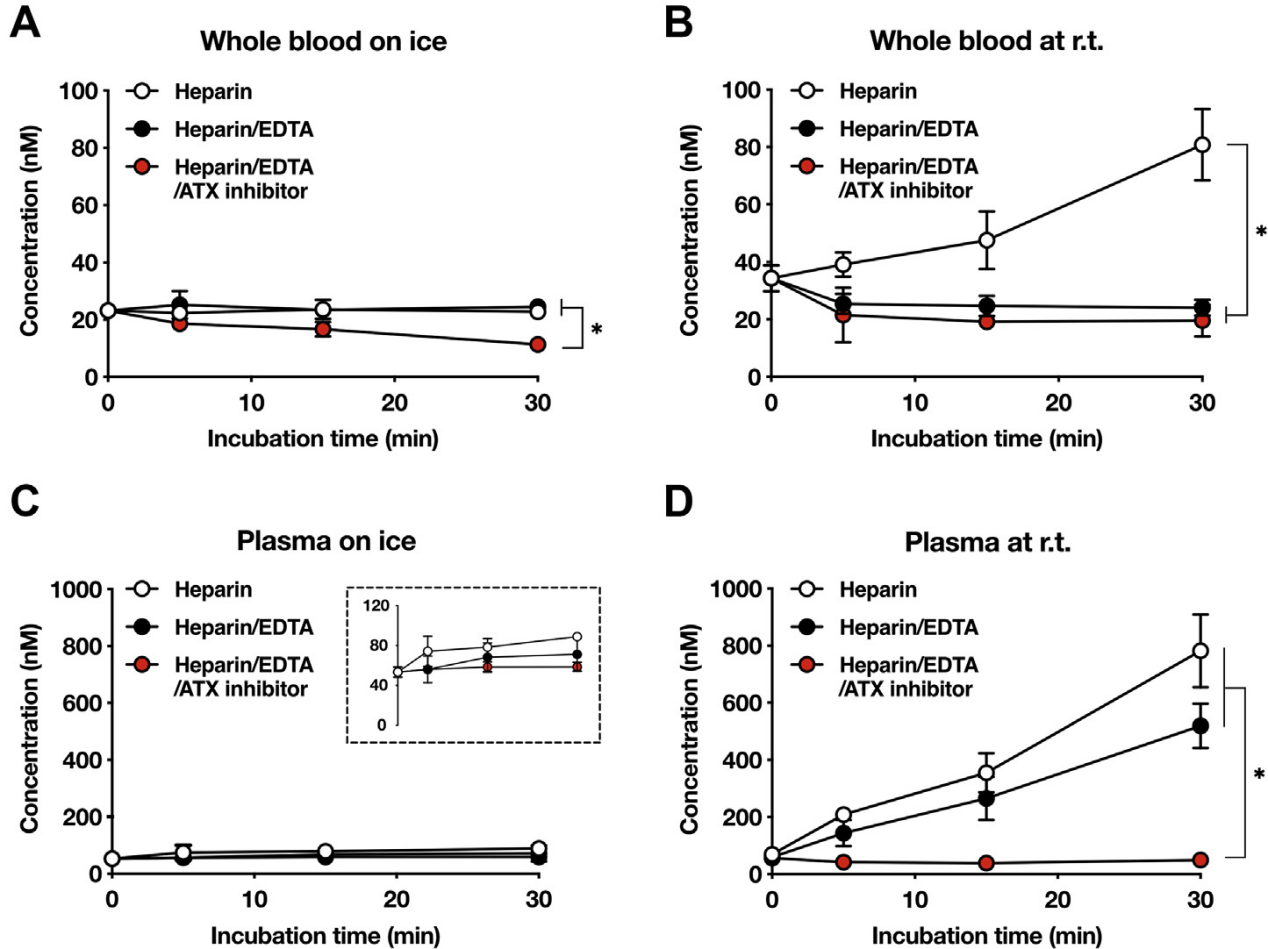
Effects of temperature and an ATX inhibitor on time-dependent changes in LPA concentration in whole mouse blood and plasma. Time-dependent changes in the concentration of total LPA in the whole blood (A, B) or plasma (C, D). Heparinized whole blood samples were added with EDTA (1 mg/ml) or ATX inhibitor ONO-8430506 (10 μM) and were kept for 30 min on ice (A) or at room temperature (r.t.) (B). Similarly, plasma samples were kept on ice (C) or at room temperature (D). At the indicated time points (0, 5, 15, and 30 min), a portion of the whole blood samples was taken and plasma was prepared (A, B). The five LPA molecular species (LPA 16:0, 18:1, 18:2, 20:4, and 22:6) in the resulting plasma samples were analyzed using LC-MS/MS for determination of the total LPA concentration. In a dashed box in (C), a graph with magnified scale is shown. Data are shown as mean ± SE (n = 3). ∗*P* < 0.05, two-way ANOVA with the Geisser-Greenhouse correction followed by Dunnett's multiple comparison test.

Changes in plasma LPA concentrations can occur in the whole blood and after plasma is isolated. We first kept mouse whole blood samples on ice with EDTA or ATX inhibitor, collected plasma by centrifugation at 4°C at different time points, and determined the LPA concentration in the resulting plasma by LC-MS/MS. We monitored the levels of the five major LPA species in blood, LPA 16:0, 18:1, 18:2, 20:4, and 22:6, to determine the total LPA concentration. When whole blood samples were kept on ice without the ATX inhibitor, the levels of total LPA in the resulting plasma did not change for at least 30 min (Fig. 2A). Of interest, however, when whole blood was kept on ice in the presence of the ATX inhibitor, the LPA levels rather decreased, indicating that LPA degradation was dominant over LPA production in the presence of the ATX inhibitor even in the low-temperature condition. To validate the result, the same experiments were performed at r.t. (Fig. 2B). A similar result was obtained for whole blood with ATX inhibitor. However, unlike the results obtained on ice (Fig. 2A), the LPA levels significantly increased in whole blood with nothing added, indicating that LPA production by ATX is dominant over LPA degradation in this condition. In whole blood with EDTA, LPA levels were also slightly decreased, suggesting that the degradation of LPA is dominant in the presence of EDTA at r.t. Taking into account an accidental temperature rise during sample handling, we decided to use EDTA containing whole blood samples and kept them on ice until plasma preparation. In addition, we decided not to add the ATX inhibitor to whole blood samples.

### A plasma handling method for accurate LPA quantitation

We then examined the artifactual changes in the concentration of LPA in plasma. Even in plasma with nothing added, the LPA levels did not change in any of the plasma samples for at least 30 min as long as the plasma samples were kept on ice (Fig. 2C). As expected, at r.t., the LPA concentration dramatically increased in a time-dependent manner in control plasma and to a slightly lesser extent in EDTA-containing plasma (Fig. 2D). However, in the presence of the ATX inhibitor, the increase in the LPA level was almost completely suppressed. We thus added the ATX inhibitor after the plasma was isolated, kept it on ice, and measured the LPA level within 30 min. These observations also demonstrated the presence of LPA-degrading activities in blood cells such as erythrocytes and lymphocytes, which were removed in the process of plasma preparation.

### Effect of platelets on LPA concentration in plasma

**Fig. 3:**
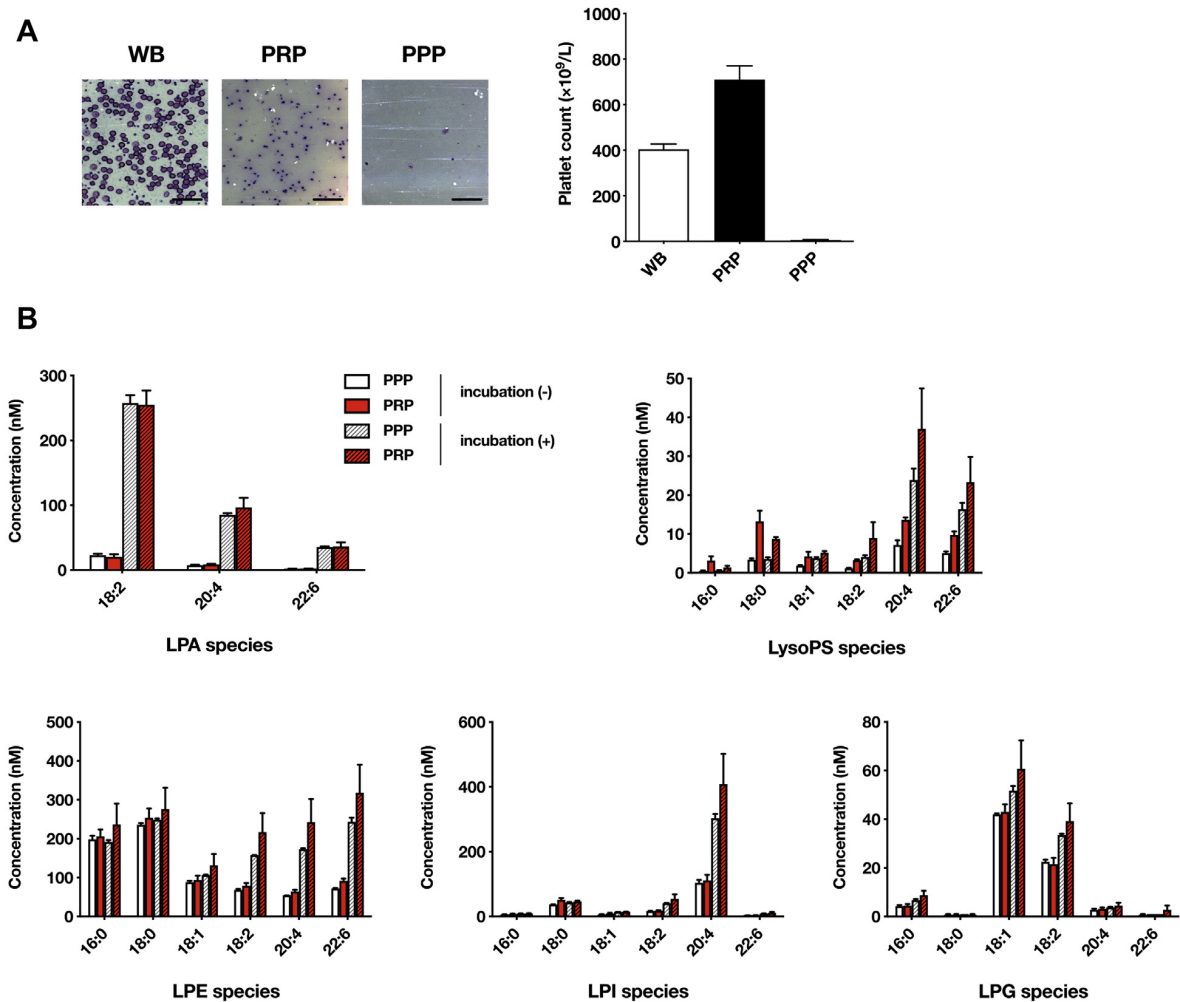
Effect of platelets on the concentration of lysophospholipids in mouse plasma. Platelet-rich plasma (PRP) and platelet-poor plasma (PPP) were prepared from mouse whole blood by centrifugation. A: Blood cells in whole blood samples (WB), PRP, and PPP (left). The numbers of platelets in PRP and PPP preparations were shown in the right. Data are shown as mean ± SE (n = 4). Bars indicate 50 μm. B: Effect of platelets and incubation time on the concentration of various lysophospholipids, including LPA, LysoPS, lysophosphatidylethanolamine, lysophosphatidylglycerol, and lysophosphatidylinositol. Incubation of plasma samples for 30 min dramatically increased the concentration of various lysophospholipid species, whereas the presence of platelets in the plasma samples had little effect on their concentration without incubation except for LysoPS. Data are shown as mean ± SE (n = 4).

As several studies have indicated that activated platelets release LPA, especially LPA 20:4 (10, 27), we next examined the contribution of platelets to the plasma LPA concentration. Both platelet-poor plasma (PPP) and platelet-rich plasma (PRP) containing EDTA were prepared from the same mice by differential centrifugation (1,500 *g* for PPP or 60 *g* for PRP). The cellular composition was quantitatively confirmed by Giemsa staining, and platelet number was determined (Fig. 3A). We found that the LPA level was almost the same in PPP and PRP. Also, the levels of other LPLs, including lysophosphatidylethanolamine, lysophosphatidylglycerol, and lysophosphatidylinositol, were not significantly different between PPP and PRP (Fig. 3B), except for LysoPS, which was noticeably higher in PRP. When PPP and PRP were incubated at r.t. for 30 min, all LPLs dramatically increased with slightly higher concentrations in PRP. These data confirmed that platelet contamination does not affect the plasma LPA concentration as long as plasma samples are kept on ice.

### Effect of freezing and thawing of plasma on LPA concentration

**Fig. 4:**
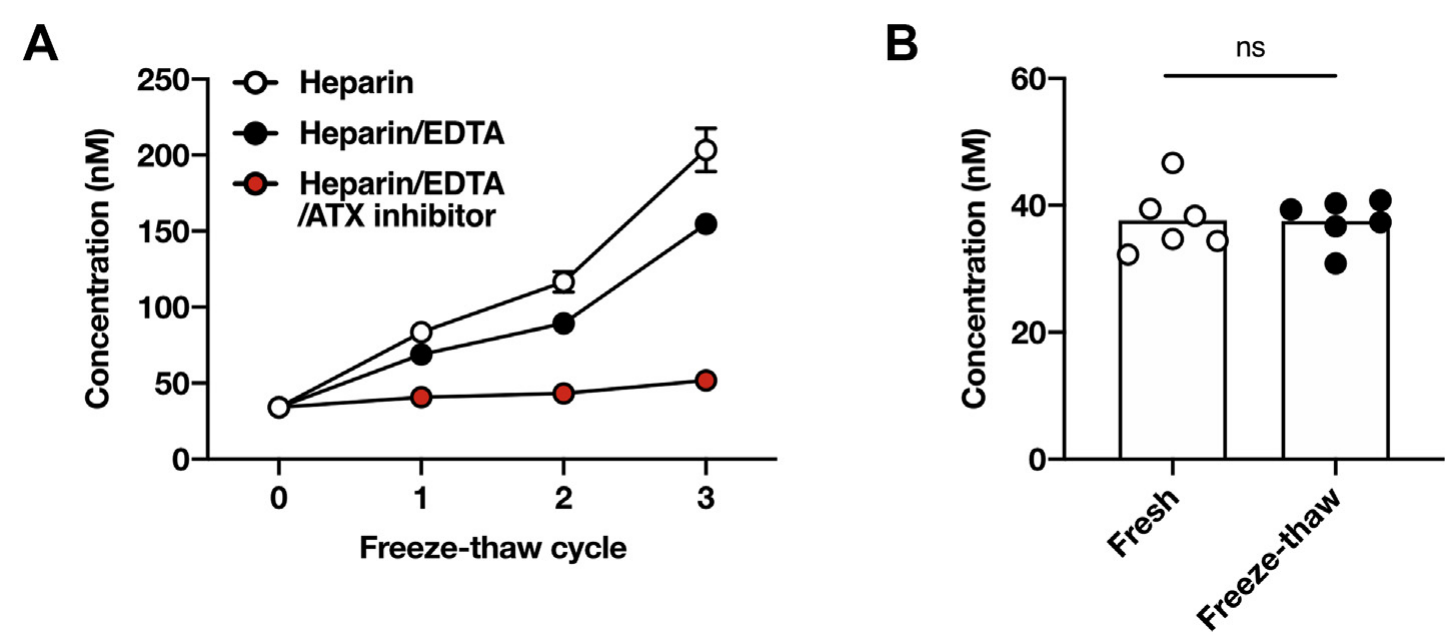
Effect of freezing and thawing of plasma on LPA concentration. A: Freshly prepared heparinized mouse plasma samples were frozen at −20°C and then thawed at the indicated times in the presence or absence of EDTA (1 mg/ml) or ONO-8430506 (10 μM), and the LPA concentration in the resulting plasma was determined by LC-MS/MS. Data are shown as mean ± SE (n = 3). B: Freshly prepared human plasma with EDTA and ONO-8430506 was subjected to one freeze-thaw cycle and then analyzed by LC-MS/MS. The bars represent the mean of six replicas, and the individual values are shown as symbols. ns, not significant, unpaired *t*-test.

We finally addressed whether freezing and thawing cycles of plasma samples affect the LPA concentration. After several cycles of freezing and thawing of plasma samples from mice, we detected an obvious increase in the LPA concentration in plasma with or without EDTA depending on the numbers of freeze-thaw cycles [fresh plasma, 34.0 ± 1.9 nM; after three cycles, 203.4 ± 24.5 nM (control) and 154.6 ± 8.5 nM (EDTA), respectively] (Fig. 4A). In the presence of EDTA and the ATX inhibitor, the artifactual elevation of the LPA concentration by repeated freezing and thawing cycles was considerably suppressed. However, a slight increase in plasma LPA was still observed (after three cycles, 51.7 ± 1.9 nM) (Fig. 4A). Thus, repeated freezing and thawing of plasma samples, even in the presence of ATX inhibitors, should be avoided.

We also examined the effect of freezing and thawing on plasma LPA concentration using human blood and confirmed that one freeze-thaw cycle was acceptable in the presence of EDTA and the ATX inhibitor (Fig. 4B).

### Validation in human blood samples

We validated the stability of LPA concentration in human blood samples using a modified method shown in supplemental Fig. S1A. Whole blood containing EDTA or EDTA/heparin was kept for 30 min on ice. The plasma sample was then prepared and kept for a further 30 min on ice in the presence of the ATX inhibitor. The results showed that the LPA concentration of the plasma prepared from blood kept on ice was comparable with that of fresh plasma (supplemental Fig. S1B). We also found that EDTA alone was sufficient as an anticoagulant to stabilize plasma LPA concentration (supplemental Fig. S1B). These data indicated the usefulness of the devised method for human blood.

### The newly devised protocol for plasma preparation

**Fig. 5:**
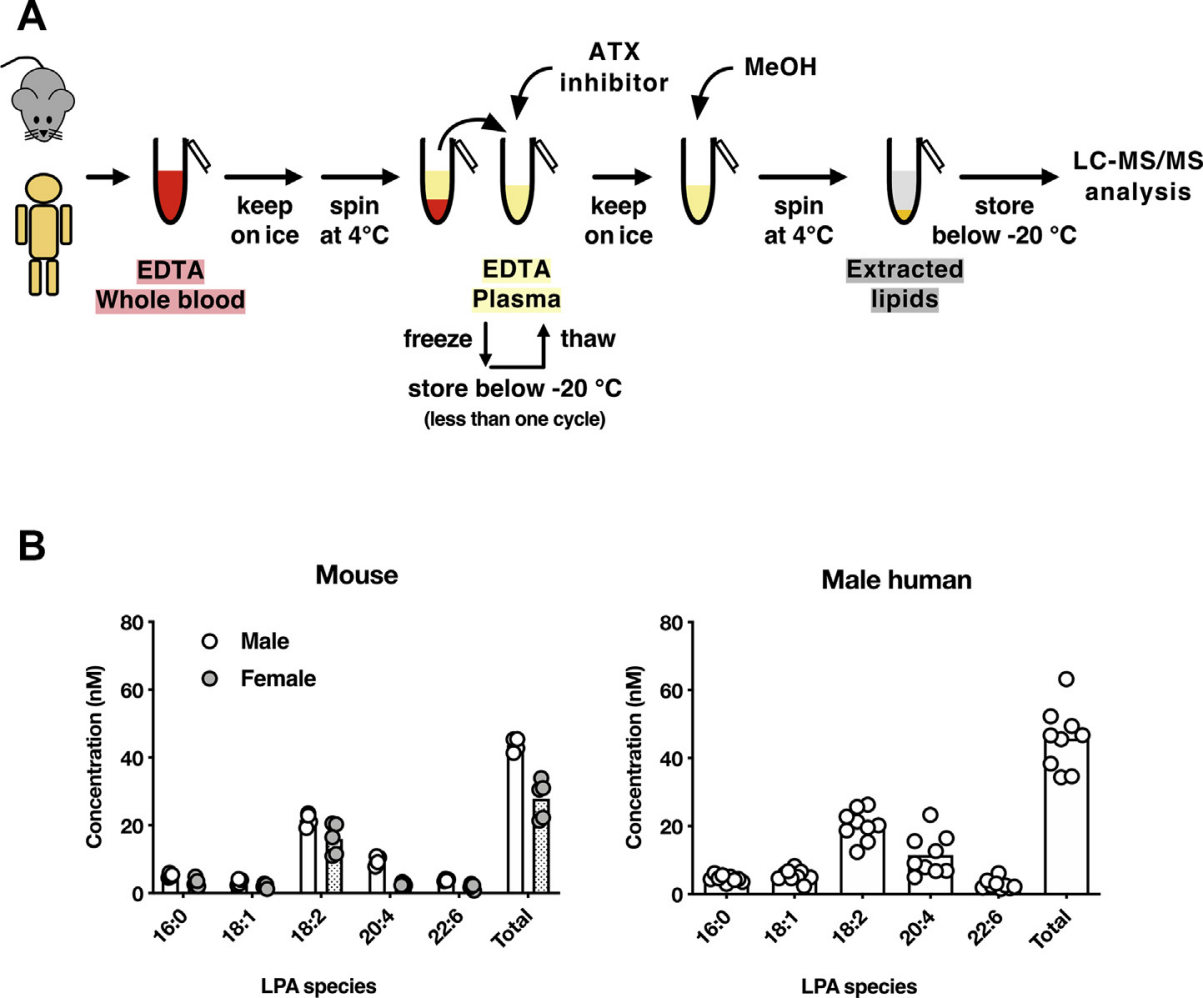
The devised plasma preparation method for precise LPA measurement and its application to mouse and human plasma. A: A simple and optimal sample preparation method for precise quantification of plasma LPA. Whole blood samples containing EDTA should be kept on ice before they are centrifuged for plasma preparation. The resulting plasma samples are also quickly kept on ice and added with an ATX inhibitor. Plasma LPA concentration is determined by LC-MS/MS after protein is removed by adding methanol to the plasma samples. B and C: The concentration of the five major LPA species (LPA 16:0-, 18:1-, 18:2-, 20:4- and 22:6) in plasma from mice (male and female) and healthy male volunteers. The bars represent the mean of four replicas, and the individual values are shown as symbols.

As we have discussed above, three conditions were found to be important in accurately determining blood LPA concentrations: (1) keeping whole blood samples at low temperatures (on ice), (2) not adding ATX inhibitors to whole blood samples, and (3) adding ATX inhibitors to the plasma after it is separated from whole blood samples. Accordingly, we developed the protocol for plasma preparation in determining accurate blood LPA concentrations, which is shown in Fig. 5A. Briefly, after EDTA whole blood samples are collected, they are kept on ice as soon as possible. Then, plasma samples are prepared by centrifuging whole blood samples at 4°C. The resulting plasma samples are quickly treated with an ATX inhibitor. The LPA concentration of the resulting plasma samples is determined by LC-MS/MS. Since the repeated freezing and thawing of plasma samples resulted in an artificial production of LPA, we did not freeze the plasma samples in the following experiments.

### LPA concentration in mouse and human plasma

Using the protocol shown in Fig. 5A, we determined the concentration of LPA species in plasma from normal mice and healthy human volunteers. In agreement with previous reports, the levels of LPA species in plasma generally follow the order: LPA 18:2 ≧ LPA 20:4 > LPA 22:6, 16:0, and 18:1. The total plasma LPA concentrations of these LPA species were similar in both mice and humans, approximately 40 nM (male) and 30 nM (female) in mice and 30–60 nM in male human (Fig. 5B), which are much lower than those reported before (0.1–40 μM). These data also suggest that plasma LPA levels had risen during sample preparation in most of the clinical research reported so far. The lower plasma LPA concentrations in female mice (Fig. 5B) can be explained by lower ATX activity in female mice (supplemental Fig. S2A), as opposed to humans (28). We also found that the plasma LPA concentrations did not significantly change between the estrus cycles in mice (supplemental Fig. S2B).

### Plasma LPA concentration is circadian regulated in mice

**Fig. 6:**
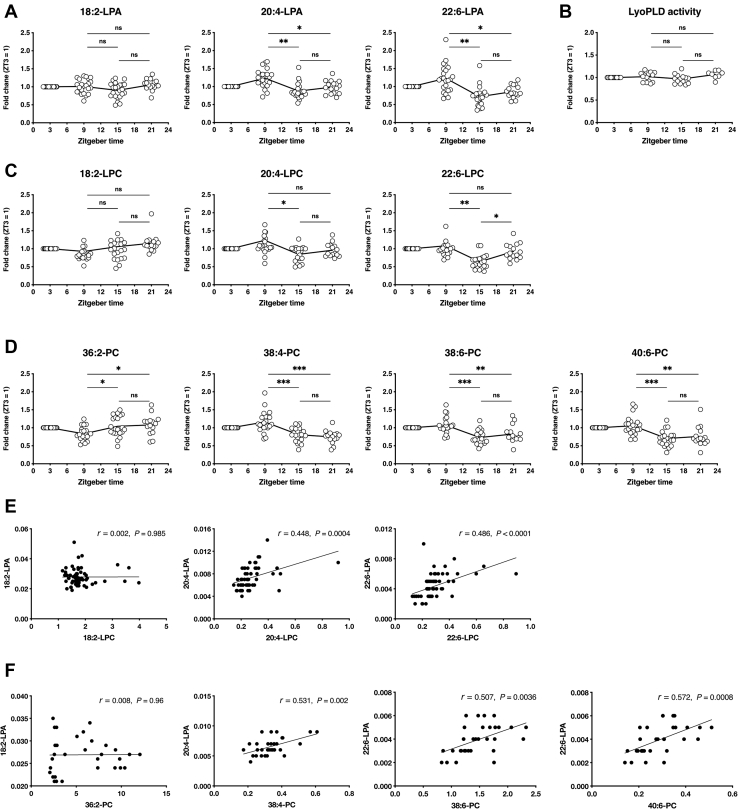
Circadian rhythms of LPA, LPC, and PC in mouse plasma. Daily fluctuation of LPA (A), ATX (B), LPC (C), and PC (D) in mouse plasma. Ad libitum fed mice were synchronized to a 12:12 h light-dark cycle. Blood samples were collected at the indicated times (shown as zeitgeber time) and analyzed according to the present method. For LPA, LPC, and PC, only phospholipids containing linoleic acid (18:2), arachidonic acid (20:4), and docosahexaenoic acid (22:6) were shown. The individual values are shown as symbols. ∗*P* < 0.05, ∗∗*P* < 0.01, ordinary one-way ANOVA followed by Tukey multiple comparison test. E and F: Correlations between LPA and LPC (E) and between LPA and PC (F).

Recent studies using lipidomic approaches have shown that hundreds of lipid species, including fatty acids and glycerophospholipids, are circadian regulated in human plasma (29, 30). For LPA, Michalczyk *et al.* (31) reported no specific circadian rhythm in human plasma, which was determined as the total concentration of various LPA species. Considering the possible artificial LPA production in the study and the fact that the study lacked data on LPA species, we investigated the temporal variation of plasma LPA in detail using the devised method (Fig. 6A). Ad libitum-fed mice were synchronized to a 12:12 h light-dark cycle, and blood was taken at four time points (ZT3, 9, 15, and 21). Among the three major LPA species, LPA 20:4 and LPA 22:6 but not LPA 18:2 showed circadian rhythms, with maximum values at ZT9 and minimum values at ZT15 (Fig. 6A). No significant differences in lysoPLD activities were observed (Fig. 6B). Of note, LPC 20:4 and 22:6, but not LPC 18:2, showed circadian rhythms similar to each corresponding LPA species (Fig. 6C, E). In addition, phosphatidylcholine (PC) species with 20:4 (PC 38:4) and 22:6 (PC 38:6 and 40:6) but not 18:2 (PC 36:2) were also circadian regulated with patterns to the patterns of the corresponding LPA species (Fig. 6D, F). These data clearly indicate that the circadian rhythms of LPA species in mouse plasma are caused by the change in the substrate supply for LPA production, not by the change in the level of the LPA-producing enzyme, ATX.

In the present study, we devised a plasma preparation method that suppresses degradation and production of LPA. In previous reports, less attention has been paid to LPA degradation. Our present results clearly show that adding ATX inhibitor to whole blood samples should be avoided since LPA degradation was dominant over LPA production in the presence of ATX inhibitor. We found that keeping the blood samples on ice was the best way to suppress LPA degradation and production, both of which were balanced in the on-ice condition at least for 30 min. As was previously suggested (15), adding an ATX inhibitor to the plasma was quite effective at suppressing LPA production after plasma was prepared. Thus, an ATX inhibitor should be added after plasma preparation. Taken together, these results indicate that keeping a balance between LPA production and degradation by putting the whole blood samples on ice is the best and the simplest way to preserve whole blood before plasma conditioning. In the present study, we also found that repeated freezing and thawing of plasma samples should be avoided even in the presence of an ATX inhibitor.

Application of the devised method revealed circadian rhythms in the plasma LPA level for the first time. Among the various LPA species, circadian rhythms were observed for certain LPA species, such as LPA with arachidonic (20:4) and docosahexaenoic (22:6) acids. Simultaneous analyses of LPC and PC species revealed the corresponding LPC and PC species containing two unsaturated fatty acids, strongly indicating that the change in the level of PC species is probably the cause of the LPA circadian rhythms. Of interest, no circadian rhythms were observed for LPA containing another polyunsaturated fatty acid, linoleic acid (18:2). It is reasonable to assume that metabolic pathways differ for each PC species. It should be stressed here that the circadian variation of LPA species could only be detected using the newly devised method, but not by conventional methods (31), demonstrating the effectiveness of this plasma conditioning method for accurate LPA measurement.

In summary, the present study developed a plasma preparation method to determine accurate plasma LPA concentrations that is applicable to human clinical studies. This method can be used to determine the LPA level in various biological fluids other than plasma (ascites, cerebrospinal fluid, saliva, urine, etc.) and to clarify the involvement of LPA in pathological states in the near future.

### Data availability

All data are contained within the article.

## Conflict of interest

The authors declare that they have no conflicts of interest with the contents of this article.
