# Supplementary material for: Suppressing postcollection lysophosphatidic acid metabolism improves the precision of plasma LPA quantification
Source: J Lipid Res. 2021 Jan 30;62:100029. doi: 10.1016/j.jlr.2021.100029 (PMC7937979; doi:10.1016/j.jlr.2021.100029)
Supplement: Supplemental Figures S1 and S2 [file mmc1.docx]

**SUPPLEMENTAL INFORMATION:**

**Preventing lysophosphatidic acid (LPA) metabolism is important for precise quantification of LPA in plasma**

Kuniyuki Kano^1,2,3^, Hirotaka Matsumoto^2^, Nozomu Kono^1^, Makoto Kurano^3,4^, Yutaka Yatomi^3,4^ and Junken Aoki^1,2,3^

1 Department of Health Chemistry, Graduate School of Pharmaceutical Sciences, University of Tokyo, 7-3-1, Hongo, Bunkyo-ku, Tokyo, 113-0033, Japan,

2 Laboratory of Molecular and Cellular Biochemistry, Graduate School of Pharmaceutical Sciences, Tohoku University, 6-3 Aoba, Aramaki, Aoba-Ku, Sendai, 980-8578, Japan,

3 AMED-LEAP, Japan Science and Technology Corporation, 4-1-8, Honcho, Kawaguchi, Saitama, 332-0012, Japan,

4 Department of Clinical Laboratory, University of Tokyo Hospital, 7-3-1 Hongo, Bunkyo-ku, Tokyo, 113-8655, Japan,

**Supplementary Figure S1: Usefulness of the devised method in human blood samples.**

(A) Experimental schemes how human plasma samples were prepared.

(B) Total LPA concentration in human plasma. LPA concentration was not significantly changed during plasma sample preparation using the devised method. The bars represent the mean of six replicas, and the individual values are shown as symbols. ns: not significant, 2-way ANOVA followed by Sidak's multiple comparison test.

**Supplementary Figure S2: The level of plasma LPA and ATX across the estrous cycle in mice.**

Plasma samples were obtained from male mice and female mice at different estrous stages (diestrus, proestrus, estrus and metestrus), and lysoPLD activity (A) and total LPA concentration (B) were determined. The bars represent the mean of replicas, and the individual values are shown as symbols. ns: not significant, **P < 0.01, one-way ANOVA followed by Tukey's multiple comparison test.
